# Rapid and efficient generation of neural progenitors from adult bone marrow stromal cells by hypoxic preconditioning

**DOI:** 10.1186/s13287-016-0409-x

**Published:** 2016-10-07

**Authors:** Kwan-Long Mung, Yat-Ping Tsui, Evelyn Wing-Yin Tai, Ying-Shing Chan, Daisy Kwok-Yan Shum, Graham Ka-Hon Shea

**Affiliations:** 1 Department of Orthopaedics and Traumatology, Li Ka Shing Faculty of Medicine, The University of Hong Kong, Pokfulam, Hong Kong; 2 School of Biomedical Sciences, Li Ka Shing Faculty of Medicine, The University of Hong Kong, Pokfulam, Hong Kong; 3 General Office, 5/F, Professorial Block, Queen Mary Hospital, Pokfulam, Hong Kong

**Keywords:** Bone marrow stromal cell, Hypoxia, Neural progenitor cell, Schwann cell, Stem cell niche

## Abstract

**Background:**

Bone marrow stromal cells (BMSCs) are attractive as a source of neural progenitors for ex vivo generation of neurons and glia. Limited numbers of this subpopulation, however, hinder translation into autologous cell-based therapy. Here, we demonstrate rapid and efficient conditioning with hypoxia to enrich for these neural progenitor cells prior to further expansion in neurosphere culture.

**Method:**

Adherent cultures of BMSCs (rat/human) were subjected to 1 % oxygen for 24 h and then subcultured as neurospheres with epidermal growth factor (EGF) and basic fibroblast growth factor supplementation. Neurospheres and cell progeny were monitored immunocytochemically for marker expression. To generate Schwann cell-like cells, neurospheres were plated out and exposed to gliogenic medium. The resulting cells were co-cultured with purified dorsal root ganglia (rat) neurons and then tested for commitment to the Schwann cell fate. Fate-committed Schwann cells were subjected to in vitro myelination assay.

**Results:**

Transient hypoxic treatment increased the size and number of neurospheres generated from both rat and human BMSCs. This effect was EGF-dependent and attenuated with the EGF receptor inhibitor erlotinib. Hypoxia did not affect the capacity of neurospheres to generate neuron- or glia-like precursors. Human Schwann cell-like cells generated from hypoxia-treated BMSCs demonstrated expression of S100β /p75 and capacity for myelination in vitro.

**Conclusion:**

Enhancing the yield of neural progenitor cells with hypoxic preconditioning of BMSCs in vitro but without inherent risks of genetic manipulation provides a platform for upscaling production of neural cell derivatives for clinical application in cell-based therapy.

**Electronic supplementary material:**

The online version of this article (doi:10.1186/s13287-016-0409-x) contains supplementary material, which is available to authorized users.

## Background

Emerging evidence has been provided for the occurrence in the bone marrow stroma of nestin-expressing neural crest derivatives critical for the development and maintenance of the bone marrow niche for hematopoietic stem cells [1–4]. In low-density non-adherent culture, this nestin-expressing subpopulation formed self-renewing clonal spheres which, following transfer to adherent culture, could be directed to differentiate into mesenchymal lineages [5]. Alternatively, we utilized adherent culture of the sphere cells to direct differentiation into Schwann cell-like cells (SCLCs) and then into fate-committed Schwann cells [6]. By seeding these bone marrow stromal cell (BMSC)-derived Schwann cells in a nerve guidance channel that bridged a critical gap in the severed sciatic nerve of a rat model, we unambiguously demonstrated the functional capacity of the derived Schwann cells for remyelination of regrowing axons in the channel [7]. The proportion of nestin-expressing neural progenitors in BMSC samples is minimal and is bound to decline with age [8]. Harvesting BMSCs from donors involves procedures that are painful, invasive, and time-consuming. Nevertheless, transplantation of BMSC-sourced progenitor cells into animal models of spinal cord injury yielded dose-dependent improvements in outcome [9]. This gave impetus to our pursuit of means to enrich for neural progenitors in the BMSC sample.

Oxygen tension represents a critical component in the maintenance of the stem/progenitor state. In vivo, neural stem cells reside in niches where oxygen tension is much lower than that of ambient air [10]. In vitro, hypoxic conditions promote proliferation and prevent apoptosis of neural stem/progenitor cells [11] while preserving or enhancing their multipotency [12, 13]. Culture of human neural progenitor cells in increased concentrations of epidermal growth factor (EGF) has a similar effect by increasing cell survival [14]. Correspondingly, upregulation of EGF receptor (EGFR) was observed among neural stem cells that had been subjected to hypoxic conditions [12]. It is unclear how neural progenitor cells located within the bone marrow niche respond to hypoxia.

We therefore aimed to determine if transient hypoxia could bring about expansion of the neural progenitor population in the sampled BMSCs and to find if this effect was EGF-dependent. As proof-of-principle, attempts were made to use neural progenitors thus expanded from human BMSC samples to generate human Schwann cells for use in cell-based therapy.

## Methods

### Isolation and culture of rat and human BMSCs

Adult rat BMSCs were prepared with modifications of that previously described [6, 7]. Briefly, bone marrow was aspirated from femurs of Sprague Dawley rats (200–250 g) and cultured in BMSC growth medium comprising of αMEM (Gibco) plus 15 % fetal bovine serum (FBS; Biosera, UK) on tissue culture plates (TPP). Unattached cells were removed 48 h later and the cultures were maintained for another 10 to 12 days with medium refreshed every 3 days. BMSCs were detached by incubation in TrypLE Express (Gibco) at 37 °C for 3 to 5 min and then centrifuged at 250 g for 5 min. Cells were resuspended in growth medium and plated onto new tissue culture plates at 50,000 cells/cm^2^. BMSCs were further passaged at a 1:2 ratio when 80 % confluency was attained. The expanded cells were monitored for immunopositivities of BMSC markers (CD90, CD73, and STRO-1) and hematopoietic progenitors (CD45-positive cells) using flow cytometry. BMSCs within 5 passages which were highly immunopositive for CD90, CD73, and STRO-1 (>90 %) but generally immunonegative for CD45 (<1 %), and were used for subsequent experiments.

Human bone marrow samples were obtained from the Department of Haematology and Bone Marrow Transplantation, Queen Mary Hospital, Hong Kong, from healthy donors with the study protocols approved by the Institutional Review Board, The University of Hong Kong (Study No. UW 10-157). Human BMSC cultures were established according to Wolfe et al. [15] with modifications. Briefly, 1 ml of bone marrow aspirate was added to 9 ml αMEM containing 15 % FBS and plated onto tissue culture plates. Unattached cells were removed 48 h later and the cultures were maintained for another 10 to 12 days with medium refreshed every 3 days. BMSCs were detached with TrypLE Express and plated onto new tissue culture plates at 50,000 cells/cm^2^. Human BMSCs were further expanded and monitored for BMSC and hematopoietic cell markers as mentioned above. Cultures within 5 passages were used for subsequent experiments.

### Hypoxia preconditioning

BMSCs were seeded at 50,000 cells/cm^2^ and maintained under normoxic conditions (21 % oxygen) in BMSC growth medium for up to 16 h. Upon reaching ~80 % confluence, BMSCs were placed in a hypoxia chamber at 1 % oxygen in BMSC growth medium for 24 h. Control cultures were maintained under normoxic conditions. Cell lysates were subjected to Western blot analysis for hypoxia-inducible factor (HIF)-1-alpha and EGFR, with β-actin serving as a loading control.

### Neurosphere culture

Neural progenitors were enriched from BMSC cultures as previously described with some modifications [6, 7]. Briefly, rat or human BMSCs from both normoxia and hypoxia treatment groups were plated onto UltraLow attachment six-well plates (Corning) at a density of 60,000 cells/well in sphere-forming medium comprising DMEM/F12 supplemented with 2 % B27 (Gibco), basic fibroblast growth factor (bFGF, 20 ng/ml; Peprotech) and EGF (Life Technologies). To evaluate the effect of EGF concentration on neural progenitor enrichment, three different concentrations (0.2, 20, and 200 ng/ml) were applied to rat BMSC cultures. The optimal EGF concentration as determined was subsequently applied to human BMSC cultures. The culture was maintained for 12 days with supplements refreshed every 3 days. Spheres with a diameter ≥50 μm after the enrichment period were counted and their diameters were assessed for comparison between the hypoxia and normoxia groups. For EGFR inhibition assays, sphere-forming medium containing 7.5 μM erlotinib was used. Spheres were assessed for abundance of nestin-positive cells.

To confirm that nestin-positive spheres derived from human BMSCs comprised neural progenitors, the spheres were plated onto tissue culture plates coated with poly-D-lysine (PDL, 20 μg/ml; Sigma-Aldrich) and laminin (10 μg/ml; Roche). Cells were allowed to differentiate in Neurobasal and DMEM/F12 media (1:1, v/v; Life Technologies) supplemented with 1 % FBS (Biosera). Cultures were maintained for 7 days with medium changed every 72 h. Cultures were then analyzed immunocytochemically for the neuronal marker, class III beta-tubulin (Tuj-1) and the astroglial marker, glial fibrillary acidic protein (GFAP).

### Schwann cell derivation and myelin formation

A schematic diagram summarizing the process of generating fate-committed Schwann cells is illustrated in Additional file 1 (Figure S1). Neurospheres derived from normoxia- and hypoxia-treated human BMSCs were differentiated into SCLCs as previously described [6, 7]. Briefly, neurospheres were plated onto PDL/laminin-coated tissue culture surface at a density of 10 spheres/cm^2^. The cultures were maintained in glial differentiation medium comprising αMEM supplemented with β-Her (100 ng/ml; Millipore), bFGF, PDGF-AA (10 ng/ml and 5 ng/ml, respectively; both from Peprotech) and 10 % FBS (Biosera, UK) for 7 days with medium replenished every 2 days. Cells were then monitored immunocytochemically for the Schwann cell markers, p75 and S100β.

Cultures with >80 % of cells immunopositive for p75 and S100β were regarded as SCLCs; these were cocultured with purified dorsal root ganglion (DRG) neurons to achieve commitment to the Schwann cell fate [6]. Briefly, dissociated DRGs (embryonic day 14-15 rat) were plated onto PDL/laminin-coated plates and then maintained in Neurobasal medium supplemented with 2 % B27 and NGF (10 ng/ml; Covance). Pulse treatment with 5-fluoro-2'-deoxyuridine and uridine (10 μg/ml each; Sigma-Aldrich) from days 2 to to 10 was performed to eliminate endogenous Schwann cells and fibroblasts in DRGs. SCLCs were then co-cultured with the purified DRG neurons in glial differentiation medium/neuron maintenance medium (1:1, v/v) for 2 weeks. By day 15, co-cultures were passaged to eliminate neurons. Subcultures of the BMSC-derived Schwann cells remained viable, showing immunopositivities for p75, S100β and the human nuclear antigen.

Schwann cells derived from normoxia- and hypoxia-treated BMSCs in co-culture with purified DRG neurons were subjected to myelin-forming conditions of DMEM/F12 and Neurobasal medium (1:1, v/v) supplemented with 2 % B27, 10 ng/ml NGF, and 50 μg/ml vitamin C (Sigma-Aldrich). The co-cultures were maintained for 15 days and then monitored immunocytochemically for myelin basic protein (MBP)-positive segments.

### Flow cytometry

All steps were performed at 22 °C unless otherwise specified. Cells were dissociated from cultures by brief treatment with TrypLE Express (37 °C) and centrifuged at 250 g for 5 min. Cells were fixed using 4 % paraformaldehyde (PFA) in phosphate-buffered saline (PBS) for 10 min and then washed with PBS. Cells were resuspended in blocking buffer comprising PBS with 3 % v/v normal goat serum (NGS; Millipore) and 0.01 % v/v Triton X-100 (Sigma-Aldrich) for 30 min. After centrifugation, cells were incubated with selected primary antibody in blocking buffer for 2 h. Thereafter, cell pellets were resuspended in appropriate secondary antibody in blocking buffer for 1 h. Cells were subjected to flow cytometric analysis with the use of BD Canto II analyzer. For each marker, at least 10,000 cells were analyzed.

Primary antibodies used were: CD90 (mouse anti-rat/human, 1:200; BD Bioscience), CD73 (mouse anti-rat/human, 1:200; BD Bioscience), STRO-1 (mouse anti-rat/human, 1:50; R&D Systems), CD45 (mouse anti-rat/human, 1:200; BD Bioscience), and nestin (mouse anti-rat/human, 1:200; BD Bioscience). Mouse isotype control (1:200; Life Technologies) was used in the control experiments. Secondary antibody used was donkey anti-mouse Alexa 488 (1:500; Life Technologies).

### Immunocytochemistry

All steps were performed at 22 °C unless otherwise specified. Cells were fixed with 4 % PFA in PBS for 10 min and then rinsed with PBS. The fixed cells were incubated in blocking buffer comprising PBS with 3 % v/v NGS and 0.1 % v/v Triton X-100 for 30 min.

Samples were incubated with selected combinations of primary antibodies overnight at 4 °C followed by approiate combinations of secondary antibodies for 60 min. Hoechst stain (Sigma-Aldrich) was used to reveal cell nuclei. In human SCLC/rat DRG co-cultures, nuclei were labeled with anti-human nuclear antigen (mouse anti-human, 1:500; Millipore) to ensure that resultant Schwann cells did not originate from DRG contaminants. Images were captured with the use of Olympus IX71 inverted fluorescence microscope fitted with an Olympus DP71 camera and Olympus analysis LS Professional imaging software. For cell counting, 10 random fields were taken and a minimum of 500 cells were counted per marker analyzed.

Primary antibodies against class III beta-tubulin (Tuj-1; mouse anti-rat/human, 1:1000; Covance), p75 (mouse anti-rat/human, 1:200; Millipore), human nuclei (mouse anti-human, 1:500; Millipore), S100β (rabbit anti-rat/human, 1:500; Dako), GFAP (rabbit anti-rat/human, 1:500; Dako), MBP (mouse anti-human, 1:500; Millipore), and neurofilament 200 ( NF200,rabbit anti-rat, 1:200; Sigma-Aldrich) were used. Secondary antibodies used were goat anti-mouse Alexa 488 and goat anti-rabbit Alexa 594 (1:400 for both; Life Technologies). Mouse and rabbit isotype controls (1:200; both Life Technologies) were used in the control experiments (Additional file 2: Figure S2). Human BMSCs and NIH/3T3 mouse fibroblast line were also used as negative controls against the neuronal marker Tuj1 and glial markers S100β, p75, and GFAP (Additional file 3: Figure S3).

### Immunoblotting

All steps were performed at 22 °C unless otherwise specified. Cells were lysed in buffer comprising TrisHCl (pH 7.4), 1 % Nonidet P-40, 0.25 % sodium deoxycholate, 150 mM NaCl, 1 mM EDTA, 1 mM PMSF, 1 μg/ml aprotinin, leupeptin, pepstatin, 1 mM Na_3_VO_4_, and 1 mM NaF. Samples were subjected to SDS/PAGE and transfer-blotted onto PVDF membrane. Membrane was blocked with 1 % BSA before incubation with the selected primary antibody at 4 °C overnight. Primary antibodies used were anti-EGFR (1:1000, mouse-anti rat/human; Santa Cruz) and anti-HIF1α (mouse anti-rat/human, 1:1000; GeneTex). The membrane was incubated with HRP-conjugated anti-mouse secondary antibody (Amersham). Bands were detected with the use of Advansta ECL (Advansta).

### Statistics

Student’s *t* test was used to determine statistically significant differences between treatment and control groups. Statistical significance was accepted at *P* < 0.05.

## Results

### Marker profiles of human and rat BMSCs

**Fig. 1:**
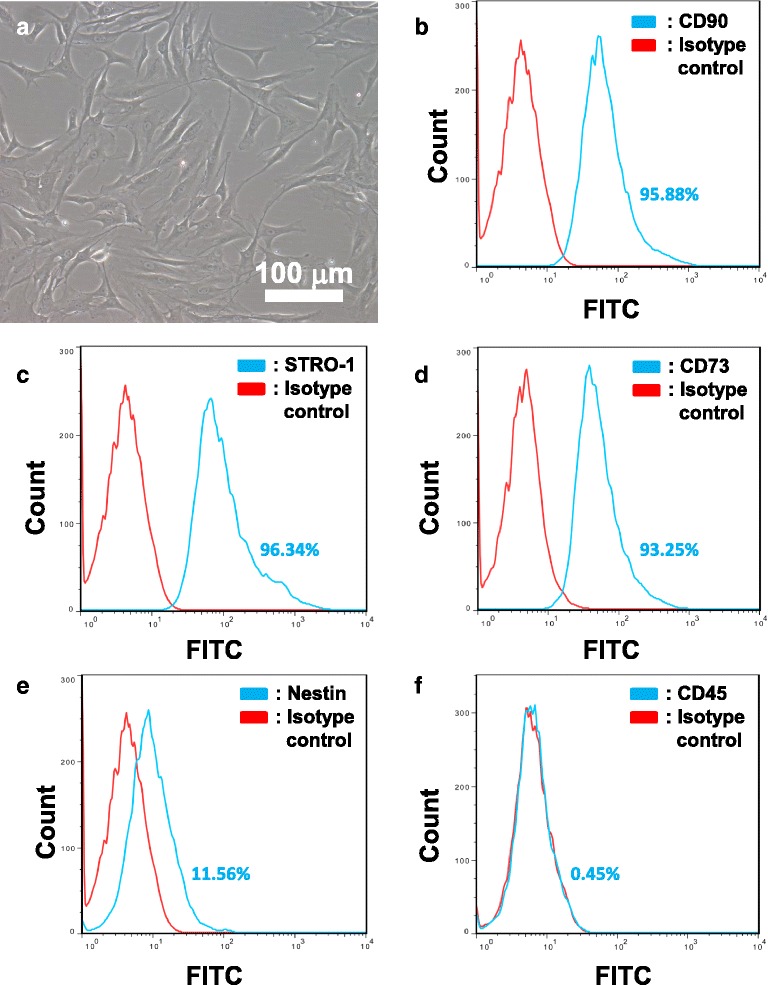
Flow cytometry analysis of human BMSCs. Human BMSCs (**a**) between passages 5 to 8 were analyzed by flow cytometry. In all panels, isotype controls are represented by *red lines*, while immunopositivity for respective cell surface markers are represented by *blue lines*. Percentages of positive cells as shown within individual panels are representative of one sample. Human BMSCs were found to be highly immunopositive for CD90 (**b**; 93.90 ± 1.77 %), STRO-1 (**c**; 93.48 ± 2.48 %), and CD73 (**d**; 91.76 ± 1.30 %). The neural stem/progenitor marker nestin was also found to be positive in a significant subpopulation of cells (**e**; 10.31 ± 1.10 %). Hardly any (<1 %) of the cells were immunopositive for the hematopoietic stem cell marker CD45 (**f**). Mean ± SD, *n* = 4

Adherent cultures of adult human and rat whole bone marrow samples were cleared of hematopoietic cells by passage 4. The resulting human BMSCs exhibited bipolar/tripolar morphology (Fig. 1a) and >90 % of them demonstrated immunopositivity for the BMSC markers CD90, STRO-1, and CD73 (Fig. 1b–d, flow cytometry data). Similarly, >90 % of the resulting rat BMSCs (Additional file 4: Figure S4A) were immunopositive for CD90, STRO-1, and CD73 (Additional file 4: Figure S4B–D, flow cytometry data). The neural stem/progenitor marker nestin was immunodetectable in approximately 10 % of human (Fig. 1e) and rat (Additional file 4: Figure S4E) BMSCs. Hardly any (<1 %) of the human (Fig. 1f) or rat BMSCs (Additional file 4: Figure S4F) were immunopositive for the hematopoietic progenitor marker CD45. These data indicated that both rat and human bone marrow cells recoverable by adhesion to tissue culture plastic were predominantly marrow stromal cells and that they include a nestin-positive subgroup. The results are consistent with findings by our group [6] and others [1, 16].

### Hypoxic preconditioning increased EGFR expression in adult BMSCs

**Fig. 2:**
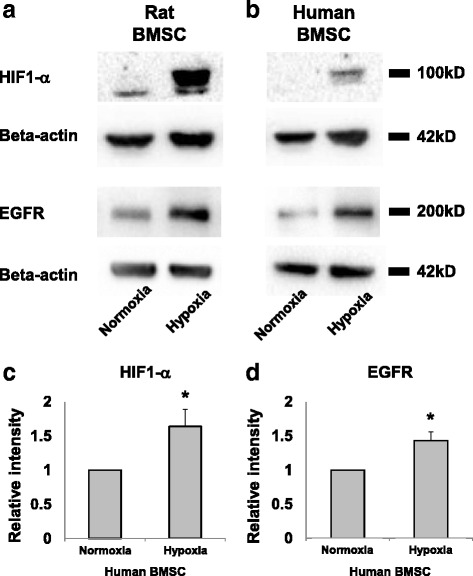
Acute hypoxia triggers upregulation of hypoxia-inducible factor-1 alpha (*HIF-1α*) and epidermal growth factor receptor (*EGFR*) in bone marrow stromal cells (*BMSCs*). Western blots of cell lysates for rat (**a**) and human BMSCs (**b**) subjected to hypoxia demonstrated increases in HIF-1α and EGFR protein expression as compared to normoxia controls. **c**, **d** Histograms to show densitometric analysis of the indicated protein bands for human BMSCs. Mean ± SD, *n* = 3; *p < 0.05

Hypoxia not only induces rapid synthesis of HIF-1α but also causes cytoplasmic HIF-1α subunits to translocate to the nucleus where they heterodimerize with HIF-1β and activate transcription of hypoxia-responsive genes [17]. As expected, HIF-1α protein levels were increased in both rat and human BMSCs subjected to hypoxia. EGFR expression was also increased (Fig. 2a and b). The increase in expression of both proteins reached statistical significance in replicates (Fig. 2c and d). Our results indicated that acute hypoxia was sufficient to upregulate the EGFR level in BMSCs.

### Hypoxic preconditioning increased the size and number of neurospheres generated from BMSCs

**Fig. 3:**
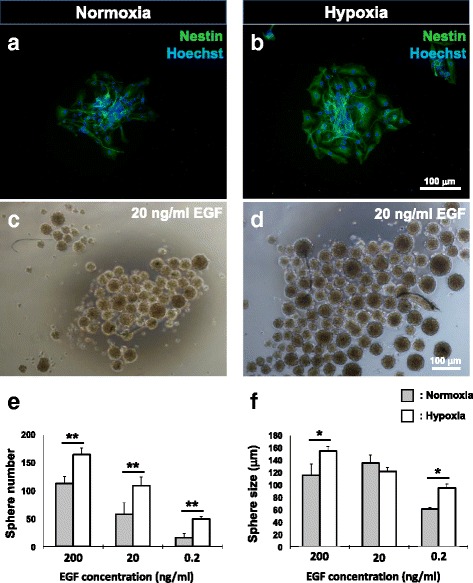
Hypoxia increases the size and number of neurospheres derived from rat BMSCs in an epidermal growth factor (*EGF*)-dependent manner. Representative images of nestin-positive rat BMSC-derived neurospheres formed following normoxia (**a**, **c**) and hypoxic preconditioning (**b**, **d**) with EGF supplementation at 20 ng / ml. **e** Hypoxic preconditioning led to an increase in sphere numbers with EGF concentrations at 200, 20, and 0.2 ng/ml. **f** A significant increase in sphere size as compared to the corresponding normoxic control was observed with EGF supplementation at 200 and 0.2 ng/ml. Mean ± SD, *n* = 4; *p < 0.05, **p < 0.01

BMSCs from rats were subjected to neurosphere culture in medium supplemented with bFGF and EGF. Consistent with our prior findings [6], resultant spheres consisted of cells that were largely immunopositive for nestin (Fig. 3a and b), suggesting the selection and expansion of neural progenitors in sphere culture. A significant increase in numbers of neurospheres was observed in hypoxia-treated BMSCs (Fig. 3c–e). To investigate the effects of EGFR upregulation on neurosphere growth, three EGF concentrations (200, 20, and 0.2 ng/ml) were applied to the cultures. The relative increase in sphere numbers after hypoxia was found to be highest with the EGF supplement at 0.2 ng/ml (300 % increase from 16 ± 8 to 50 ± 4 spheres), followed by 20 ng/ml (90 % increase, from 58 ± 21 spheres to 109 ± 15 spheres), and then 200 ng/ml (45 % increase, from 113 ± 13 spheres to 165 ± 12 spheres). Hypoxia also resulted in an increase in sphere size (Fig. 3f), an effect which was found to be significant with the EGF supplement at 0.2 ng/ml and 200 ng/ml. Overall, the sphere number and size increased with the concentration of EGF supplement in the culture medium. The upregulation of EGFR in response to hypoxia suggests that increased sensitivity towards EGF ligands is a potential mechanism.

**Fig. 4:**
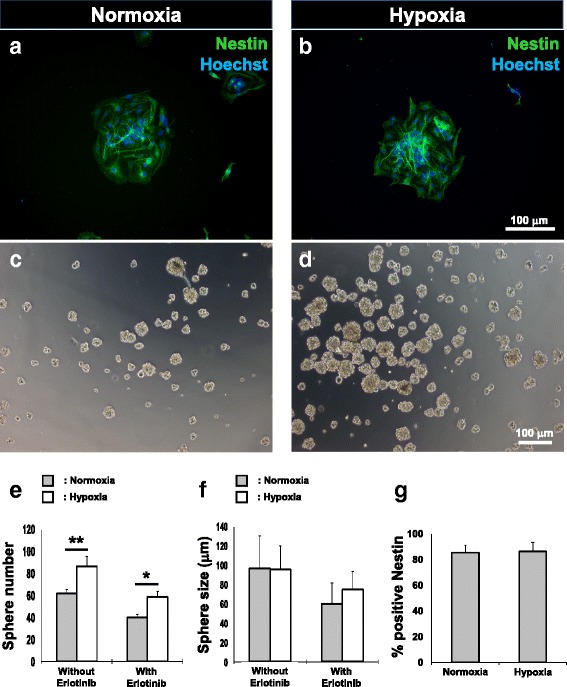
Hypoxia enriches for neural progenitors in human BMSCs. Representative images of human BMSC-derived neurosphere formation following normoxia (**a**, **c**) and hypoxic preconditioning (**b**, **d**). Numbers of neurospheres significantly increased in response to hypoxia (**e**). A reduction in sphere numbers was observed in both groups with the addition of erlotinib. However, hypoxia-treated human BMSCs yielded significantly more spheres in the presence of the EGFR inhibitor. Diameter of spheres remained similar irrespective of normoxia/hypoxia preconditioning or test/control treatment with Erlotinib (**f**). Numbers of nestin-positive cells within neurospheres were similar amongst both treatment groups (**g**). Mean ± SD, *n* = 8 for sphere counting, *n* = 4 for quantification of nestin-positive cells; *p < 0.05, **p < 0.01

Ultimately, our aim was to find a means of fostering an increase of neural progenitors in human BMSC samples for utility in autologous cell therapy. Within the range of EGF supplementation tested, 20 ng/ml approximates to that used in cultures of human neural progenitor cells [18]. We therefore utilized this concentration in subsequent neurosphere enrichment and inhibition studies with human BMSCs. Again, neurospheres derived from both hypoxia- and normoxia-treated human BMSCs consisted of cells, 80 % of which were nestin-positive (Fig. 4a, b, and g). Hypoxia-treated human BMSCs generated 40 % more spheres than the normoxic control group (62 ± 4 vs. 87 ± 9 spheres; Fig. 4c–e). The addition of the EGFR inhibitor erlotinib to the sphere cultures reduced the numbers of spheres in both hypoxia- and normoxia-treated cultures (Fig. 4e) while not significantly affecting sphere size (Fig. 4f). We again infer that signaling via EGFR is important in determining the numbers of neurospheres attainable via hypoxic treatment of BMSCs. However, under our culture conditions, the hypoxic response was still apparent in the presence of erlotinib.

### Neurospheres generated following hypoxic preconditioning contain multipotent neural progenitors

**Fig. 5:**
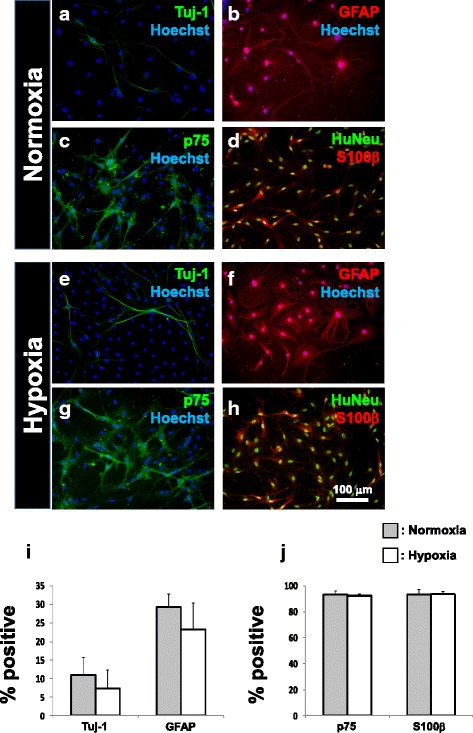
Human BMSC-derived neurospheres can generate neuron-like and glia-like cells. Cells that exited from adherent cultures of neurospheres in low-serum medium differentiated into class III beta-tubulin (*Tuj-1*) expressing neuron-like cells (**a**, **e**) as well as glial fibrillary acidic protein (*GFAP*)-expressing glia-like cells (**b**, **f**). The Tuj-1- and GFAP-positive fractions did not differ significantly between the normoxia- and hypoxia-treated groups (**i**). Adherent cultures of neurospheres in glia-inducing medium fostered differentiation of SCLCs, showing bipolar morphology, and immunopositivity for the Schwann cell markers p75 (**c**, **g**) and S100β (**d**, **h**). Numbers of p75- and S100β-positive cells derived from normoxia- and hypoxia-treated BMSCs did not differ (**j**). Mean ± SD, *n* = 4 for neuronal/glial differentiation, *n* = 3 for SCLC differentiation; *p < 0.05, **p < 0.01. *HuNeu* human nuclear antigen

To confirm that neural progenitors enriched from human BMSCs were multipotent, neurospheres from both normoxic and hypoxic treatment groups were plated onto PDL/laminin-coated culture plates and subjected to culture in low serum concentrations to allow for differentiation. Both Tuj-1-positive neuron-like cells showing branching neurites (Fig. 5a and e) and GFAP-positive glia-like cells showing radial processes (Fig. 5b and f) could be seen among cells that exited from the spheres. No significant difference was observed for the percentage of cells positive for Tuj-1 or GFAP between the normoxia and hypoxia groups (Fig. 5i). Neurosphere-derived cells from both normoxic (Fig. 5c and d) and hypoxic treatment groups (Fig. 5g and h) maintained for 7 days in glial differentiation medium yielded SCLCs, with >90 % of cells being immunopositive for the Schwann cell markers p75 and S100β [6, 19]. Again, no significant differences were observed in the percentage of cells positive for p75 or S100β between these groups (Fig. 5j). Therefore, neurospheres generated from human BMSCs following hypoxia treatment consisted of multipotent progenitors with differentiation potential towards neuron-like and glia-like fates, but hypoxia did not bias differentiation towards either lineage as has been reported [29].

### Myelin-forming Schwann cells can be generated from hypoxia-treated BMSCs

**Fig. 6:**
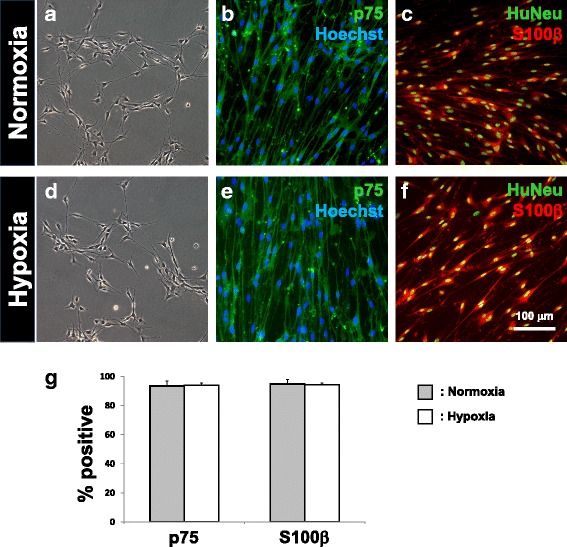
Derivation of fate-committed Schwann cells by co-culture of human SCLCs with purified rat DRG neurons. Fate-committed Schwann cells were generated following 2 weeks of co-culture between human SCLCs and purified rat DRG neurons. These cells were spindle-shaped (**a**, **d**), as well as immunopositive for p75 (**b**, **e**) and S100β (**c**, **f**). Expression of human nuclear antigen (*HuNeu*) demonstrates that these Schwann cells were not contaminated by cells originating from rat DRGs. Numbers of p75- and S100β-immunopositive cells did not show statistical difference when comparing between normoxic and hypoxic treated groups (**g**). Mean ± SD, *n* = 3; *p < 0.05, **p < 0.01

**Fig. 7:**
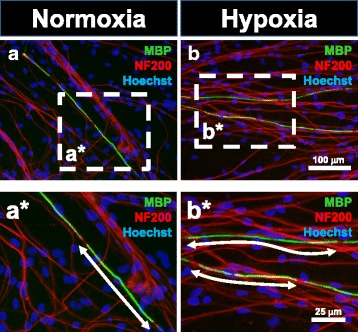
Formation of myelin basic protein (*MBP*)-positive myelin segments by human BMSC-derived Schwann cells. Schwann cells derived from normoxic (**a**) and hypoxic treatment groups (**b**) formed MBP-positive myelin segments along NF200-expressing axons of DRG neurons. Individual myelin segments are indicated in enlarged images (a*, b*)

To test if signaling from rat DRG neurons could drive human BMSC-derived SCLCs to fate commitment as we reported for rat BMSC-derived SCLCs [6], the human BMSC-derived SCLCs were co-cultured with neurons purified from rat DRG. After 15 days of co-culture, cells with bi-/tripolar morphology like those of Schwann cells (Fig. 6a and d) were detectable for both normoxic and hypoxic treatment groups. Over 90 % of cells were immunopositive for the Schwann cell markers p75 and S100β, even after withdrawal of gliogenic factors from the culture medium and passaging to remove DRG neurons (Fig. 6b, c, e and f). Contrary to the transient phenotype that is characteristic of SCLCs, persistence of marker expression indicates the progress to maturation and fate commitment in the human bone marrow-derived Schwann cells. As proof-of-principle, the Schwann cells so derived from both treatment groups were further co-cultured with rat DRG neurons and with ascorbic acid supplementation to stimulate transition into the myelination phenotype [20]. Schwann cells derived from human BMSCs of both treatment groups were thus shown to generate MBP-positive segments along the NF200-positive axons of purified DRG neurons (Fig. 7a–d). Our results support that, subsequent to hypoxic treatment to increase numbers of neural progenitors in both human and rat BMSC samples, there is potential to generate myelin-forming Schwann cells.

## Discussion

Our results demonstrate that transient exposure of BMSCs to hypoxia results in increases in the number of spheres comprising nestin-expressing progenitor cells as expanded from both rat and human samples. This coincides with upregulation in EGFR expression among the BMSCs, and increased sensitivity of the cells to EGF during expansion of the cells in sphere-forming culture. Given an adherent substratum and glia-inducing factors in the culture medium, cells on exit from the sphere cells could be directed to differentiate into SCLCs. Subjecting the SCLCs to co-culture with DRG neurons committed them to the Schwann cell fate, as reported for rat cells [6] and shown here for human cells. With ascorbic acid supplemented into the co-culture, the potential of the derived Schwann cells for myelination could be demonstrated in vitro. Our findings indicate that hypoxic preconditioning of BMSCs in vitro followed by sphere-forming culture is effective and efficient for enrichment of neural progenitors in the sample. This strategy is potentially applicable to neural progenitors harbored in other tissue samples. These neural progenitors can represent a source of autologous cells for the purpose of cell-based therapy in nerve injury, demyelinating disorders, and neurodegenerative diseases.

Cell-based therapy necessitates the isolation of stem/progenitor cells, a period of in vitro expansion, differentiation of precursors into relevant cell types, and finally transplantation. The focus of our previous work was on the directed differentiation of BMSCs into fate-committed Schwann cells [6] prior to transplantation in the injured peripheral [7] and central nervous system [21]. Meta-analysis of results in animal models of traumatic spinal cord injury demonstrates that ex vivo differentiation of stem cells prior to transplantation, as well as increasing the total numbers of cells transplanted, are pertinent to improving outcomes [9]. Here, our efforts to enhance clinical utility address the need to expand upon a small pool of neural precursor cells prior to directed differentiation. In vivo, the BMSC niche is located amongst perivascular tissues [3, 5, 8]. Despite being in the vicinity of blood vessels, cells are subject to lowered oxygen tensions and therefore culture in 21 % oxygen represents a hyperoxic milieu. In mesenchymal stem cells (MSCs), hypoxia has indeed been successfully utilized as a means of enhancing proliferation and multilineage potential for clinical utility, although variations in cell source and culture conditions have led to differing results [22]. Similar to our present findings, transplantation of hypoxia-preconditioned BMSCs into an animal stroke model has enhanced neurogenesis [23]. Our approach of expanding the neural progenitors sampled from bone marrow and the subsequent use of culture conditions to direct differentiation into mature neurons and glia has the advantage of being free from genetic reprogramming and the inherent risks associated with pluripotent stem cell-based therapies [24]. MSCs that are subject to prolonged hypoxia exhibit genetic integrity and do not demonstrate tumorigenicity [25]. We foresee the use of autologous Schwann cells generated in this manner to be a vital component of nerve guidance channels, superseding the need to sacrifice a nerve graft [6, 7]. Furthermore, transplantation of neural precursors has also progressed to clinical trials; for example, in amyotrophic lateral sclerosis [26].

When neural precursor cells are isolated and cultured under hypoxic conditions, an increase in cell proliferation occurs in combination with a decrease in apoptosis [11]. Accordingly, our results are compatible with this previous report as numbers and relative sizes of neurospheres reflect cell turnover. The HIF family of transcriptional factors are master regulators of the hypoxic response. In our findings, hypoxia apparently triggered upregulation of both HIF-1α and EGFR. Hypoxia-driven proliferation has been attributed to HIF-1α in embryonic neural progenitors. Alternatively, marked elevation of EGFR has been reported in neural progenitors located at the subventricular zone subsequent to perinatal hypoxia/ischemia [12]. Furthermore, hypoxia led to elevated expression of heparin-binding EGF [27]. Distinct populations of EGF-responsive and bFGF-responsive neural precursors exist within the embryonic telencephalon [28]. Upon reaching maturity, neural stem cells are ultimately able to proliferate and retain multipotency when cultured in either EGF or bFGF. The presence of bFGF within the culture medium possibly accounts for the presence of neurospheres despite EGFR inhibition.

Our protocol utilizes a period of preconditioning whereby plated BMSC are subjected to hypoxia prior to neurosphere culture. Conversely, when adipose tissue-derived MSCs were cultured under hypoxic conditions during sphere formation, there was neither change in sphere size nor number despite an increased predilection for neurogenesis [29]. It is noteworthy that in glioblastoma cells cultured as spheroids, the core already constitutes a hypoxic microenvironment which is able to induce EGFR upregulation [28]. It is therefore not unexpected that hypoxia conferred no additional benefit via the EGFR/EGF signaling axis when neurospheres as opposed to two-dimensional BMSC cultures were exposed to lowered oxygen tensions.

## Conclusion

Hypoxic preconditioning of BMSC samples is a simple and efficient means of triggering increases in the nestin-expressing subpopulation of BMSCs prior to expansion in sphere-forming culture followed by directed differentiation along the neural lineage. As opposed to utilizing induced pluripotent stem cells (iPSCs) or direct reprogramming to differentiated cell types, this approach avoids genetic manipulation and its associated risks. Neural precursors and their differentiated cell progeny generated via this platform can be utilized in autologous cell therapy. Potentially, hypoxic preconditioning can be applied to stem/progenitor cells recovered from other accessible MSC niches such as skin and adipose tissue.

## Additional files

Additional file 1:
**Figure S1.** Work flow for the derivation of fate-committed Schwann cells from BMSCs. BMSCs isolated by means of adherence to tissue culture plastic were dissociated and maintained for 12 days in the presence of EGF/bFGF on low-attachment culture plates. Thereafter, resultant neurospheres were plated onto PDL/laminin-coated culture plates and differentiated into Schwann cell-like cells (SCLCs) in glial differentiation medium containing β-heregulin, bFGF, and PDGF for 7 days. In order to direct SCLCs to fate commitment, they were co-cultured with purified and partially dissociated dorsal root ganglia (DRG) neurons for a further 15 days. (PDF 159 kb)
Additional file 2:
**Figure S2.** Isotype controls for immunocytochemistry. (A, B) Mouse and rabbit isotype control antibody-stained neurospheres derived following normoxic and hypoxic treatment, respectively. (C, D) Mouse and rabbit isotype control antibody-stained SCLCs derived from normoxia- and hypoxia-treated BMSCs, respectively. (E, F) Mouse and rabbit isotype control antibody-stained fate-committed Schwann cells generated from normoxia- and hypoxia-treated BMSCs, respectively. (PDF 211 kb)
Additional file 3:
**Figure S3.** Negative controls for neural marker immunocytochemistry. Immunocytochemistry performed on 3T3 cells and human BMSCs as negative controls against the neuronal marker Tuj-1 (A, B) and glial markers GFAP (C, D), p75 (E, F), and S100β (G, H) demonstrated an absence of expression. (PDF 478 kb)
Additional file 4:
**Figure S4.** Flow cytometry analysis of rat BMSCs. Rat BMSCs (A) between passage number 5 and 8 were analyzed by flow cytometry. In all panels, isotype controls are represented by red lines, while immunopositivity for respective cell surface markers are represented by blue lines. Percentages of positive cells as shown within individual panels are representative of one sample. Immunopositivity for CD90, STRO-1, and CD73 in isolated rat BMSCs were 95.70 ± 1.21 %, 95.91 ± 1.86 %, and 92.80 ± 1.46 %, respectively (B–D). Immunopositivity for nestin was 11.31 ± 1.25 % (E). Immunopositivity towards the hematopoietic progenitor marker CD45 was negligible (F). Mean ± SD, *n* = 4. (PDF 288 kb)

## Abbreviations

bFGF: Basic fibroblast growth factor
BMSC: Bone marrow stromal cell
DRG: Dorsal root ganglion
EGF: Epidermal growth factor
EGFR: Epidermal growth factor receptor
FBS: Fetal bovine serum
GFAP: Glial fibrillary acidic protein
HIF: Hypoxia-inducible factor
MBP: Myelin basic protein
MSC: Mesenchymal stem cell
NGS: Normal goat serum
p75: Low-affinity nerve growth factor receptor
PBS: Phosphate-buffered saline
PDL: Poly-D-lysine
PFA: Paraformaldehyde
SCLC: Schwann cell-like cell
Tuj-1: Class III beta-tubulin
