# Supplementary material for: Rapid and efficient generation of neural progenitors from adult bone marrow stromal cells by hypoxic preconditioning
Source: Stem Cell Res Ther. 2016 Oct 7;7:146. doi: 10.1186/s13287-016-0409-x (PMC5055711; doi:10.1186/s13287-016-0409-x)
Supplement: Additional file 1: — Figure S1. Work flow for the derivation of fate-committed Schwann cells from BMSCs. BMSCs isolated by means of adherence to tissue culture plastic were dissociated and maintained for 12 days in the presence of EGF/bFGF on low-attachment culture plates. Thereafter, resultant neurospheres were plated onto PDL/laminin-coated culture plates and differentiated into Schwann cell-like cells (SCLCs) in glial differentiation medium containing β-heregulin, bFGF, and PDGF for 7 days. In order to direct SCLCs to fate commitment, they were co-cultured with purified and partially dissociated dorsal root ganglia (DRG) neurons for a further 15 days. (PDF 159 kb) [file 13287_2016_409_MOESM1_ESM.pdf]

Derivation of fate-committed Schwann cells from BMSCs

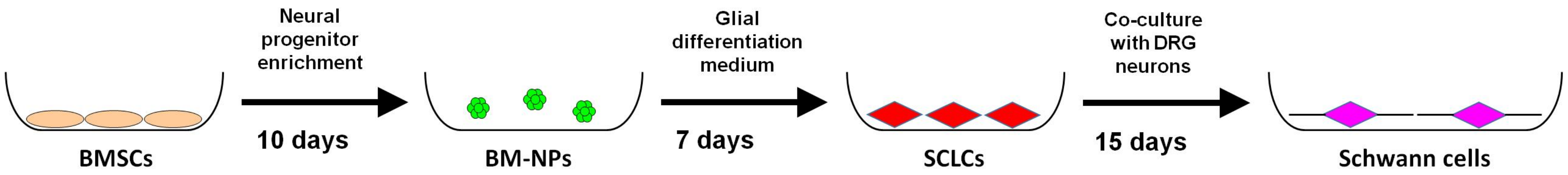

BMSC = bone marrow stromal cell
BM-NP = bone marrow-derived neural progenitors
SCLC = Schwann cell-like cell
