# Supplementary figures and images for: Rapid and efficient generation of neural progenitors from adult bone marrow stromal cells by hypoxic preconditioning
Source: Stem Cell Res Ther. 2016 Oct 7;7:146. doi: 10.1186/s13287-016-0409-x (PMC5055711; doi:10.1186/s13287-016-0409-x)

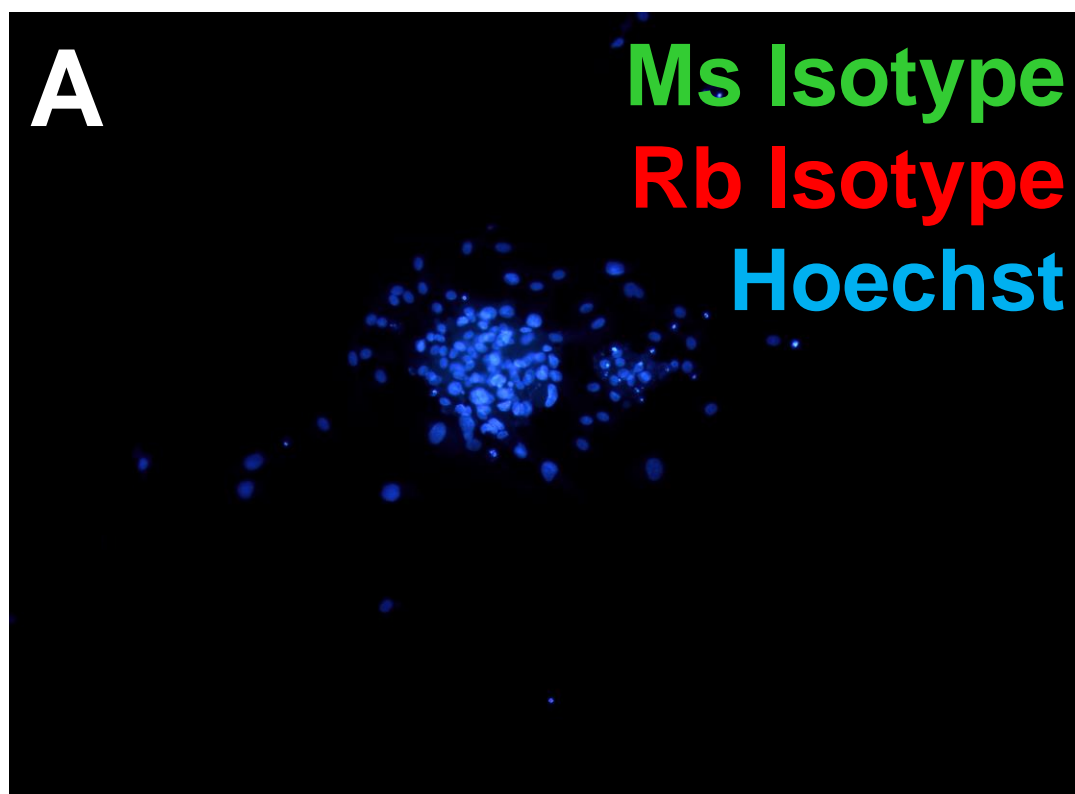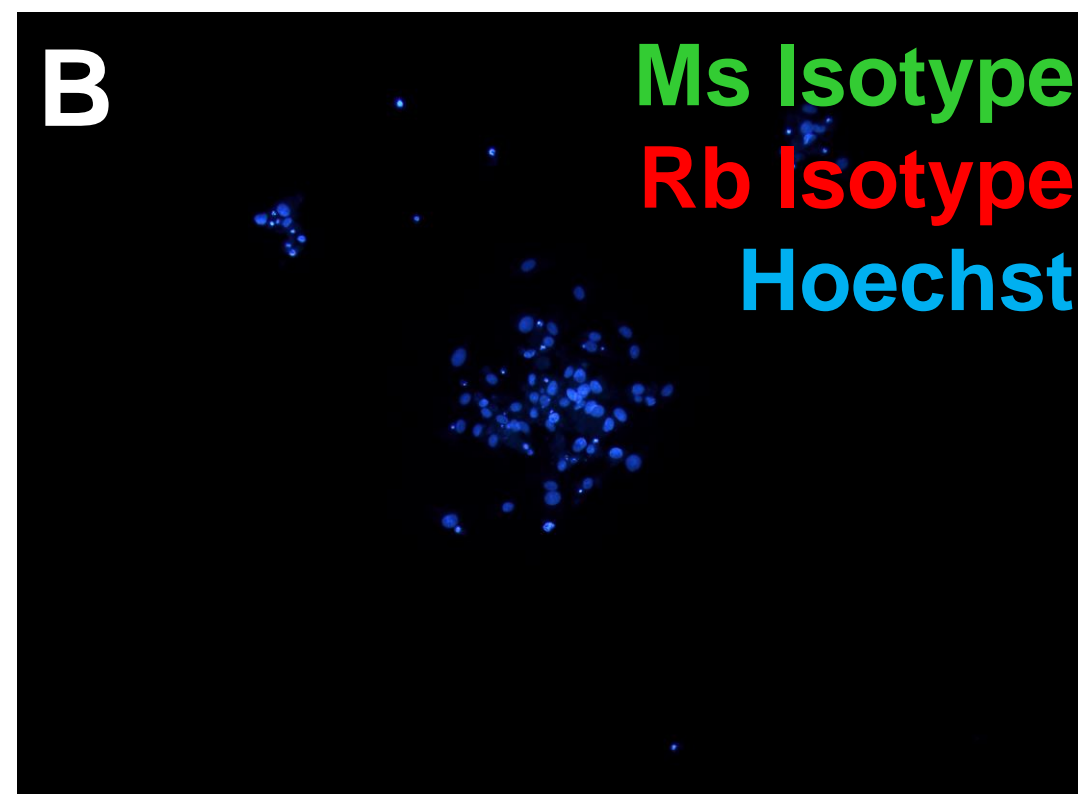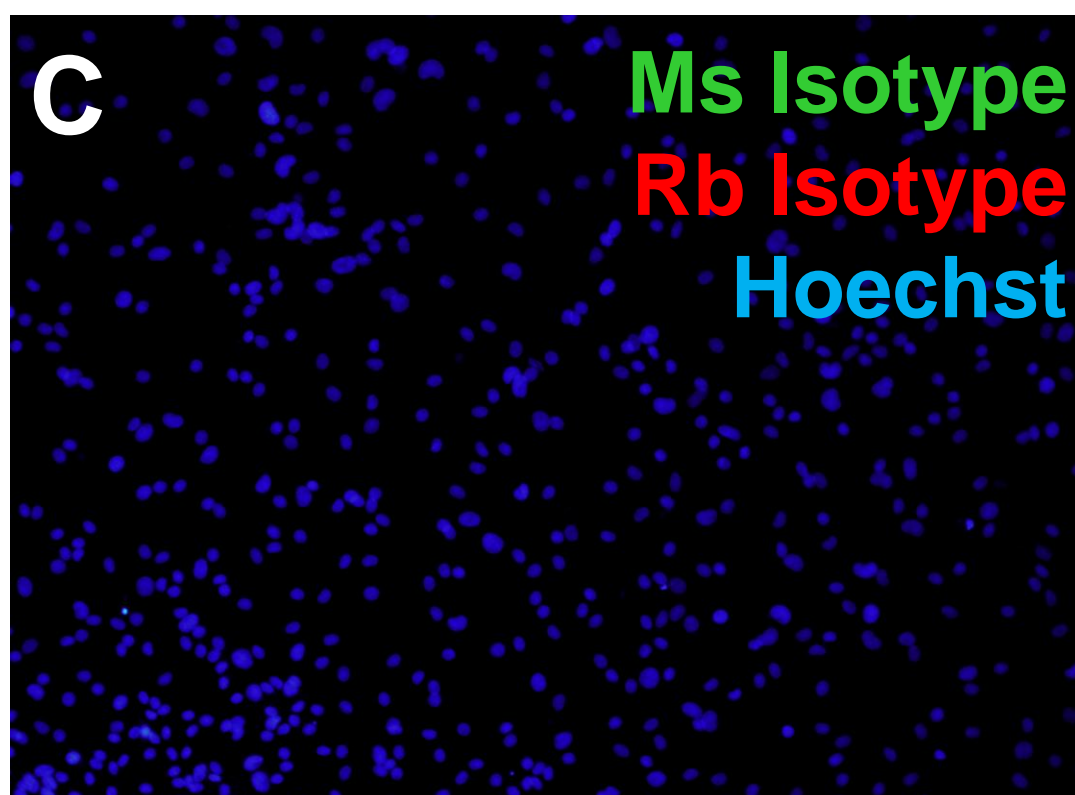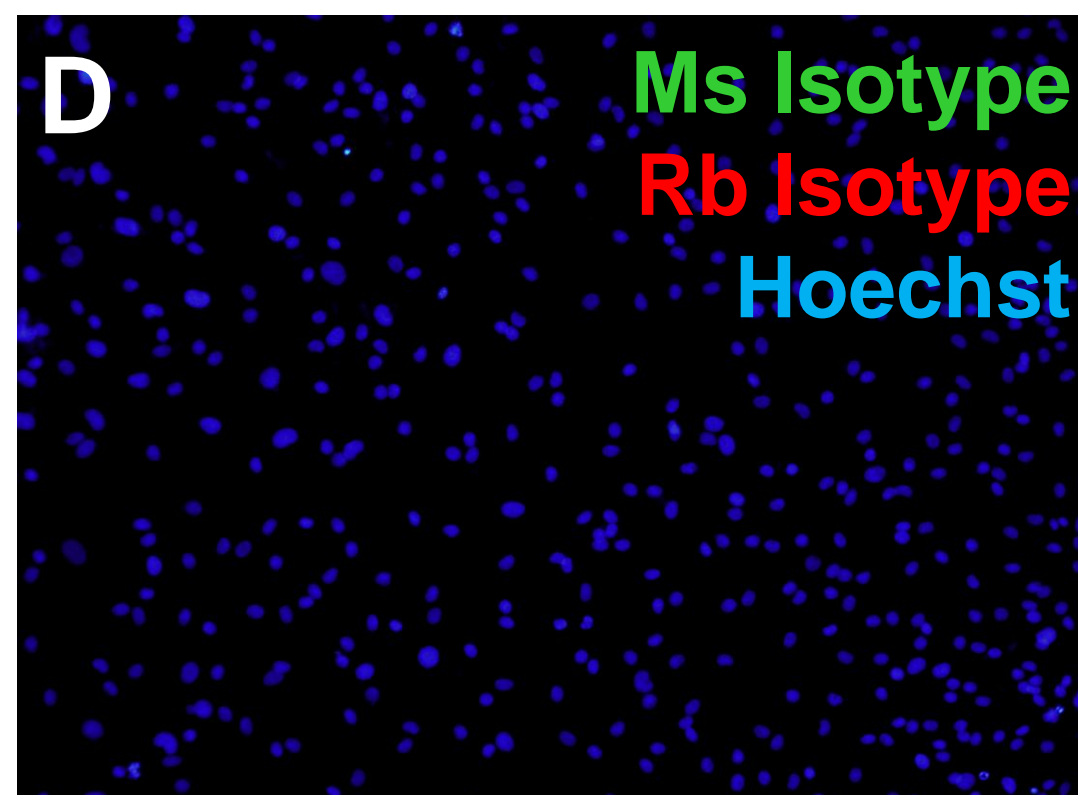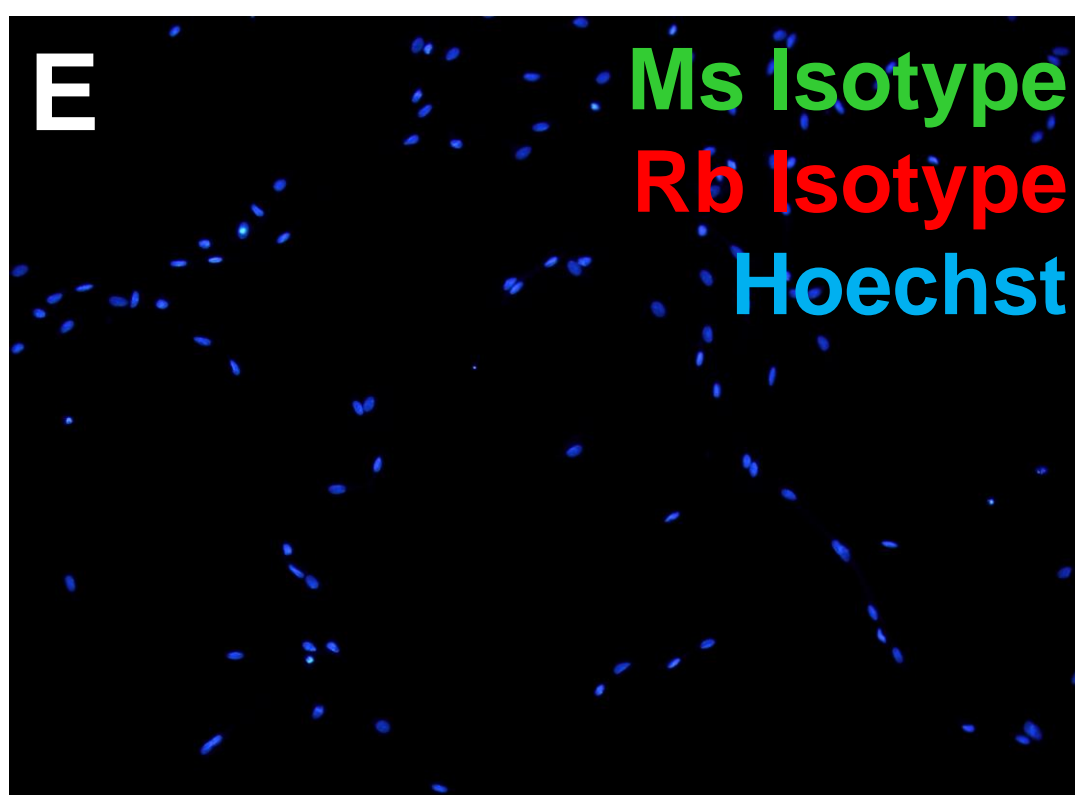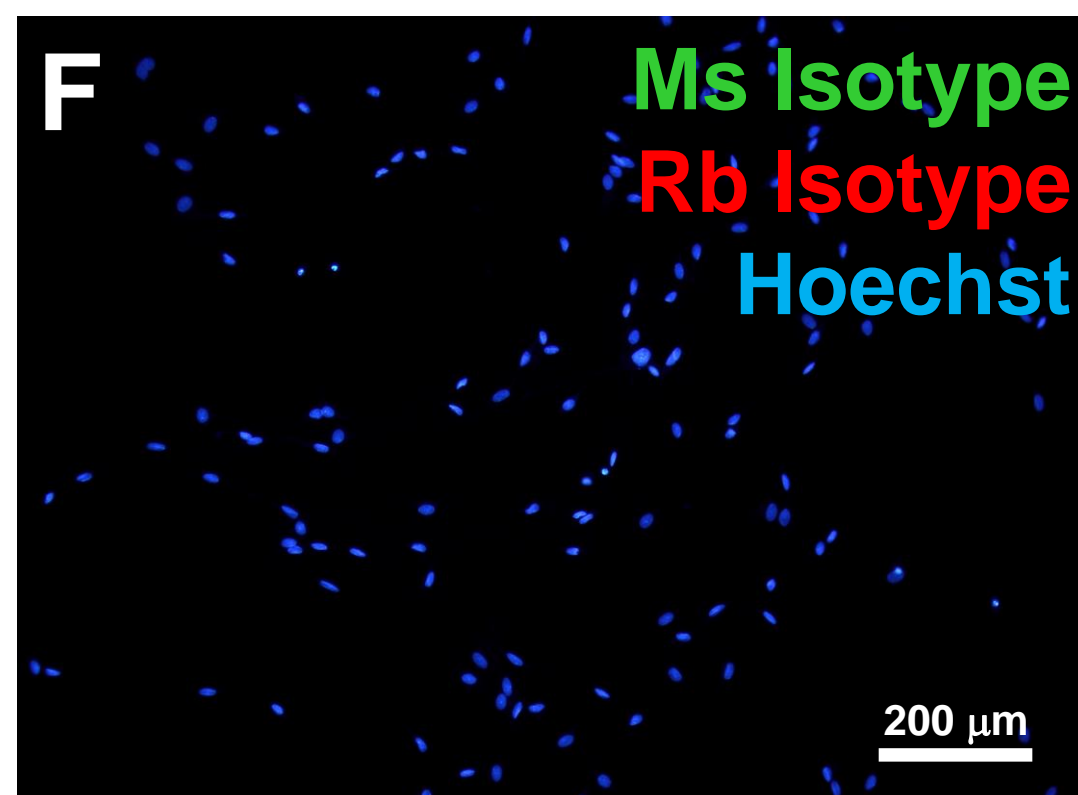

Supplement: Additional file 2: — Figure S2. Isotype controls for immunocytochemistry. (A, B) Mouse and rabbit isotype control antibody-stained neurospheres derived following normoxic and hypoxic treatment, respectively. (C, D) Mouse and rabbit isotype control antibody-stained SCLCs derived from normoxia- and hypoxia-treated BMSCs, respectively. (E, F) Mouse and rabbit isotype control antibody-stained fate-committed Schwann cells generated from normoxia- and hypoxia-treated BMSCs, respectively. (PDF 211 kb) [file 13287_2016_409_MOESM2_ESM.pdf]

## 3T3 cells

## HuBMSCs

Tuj-1

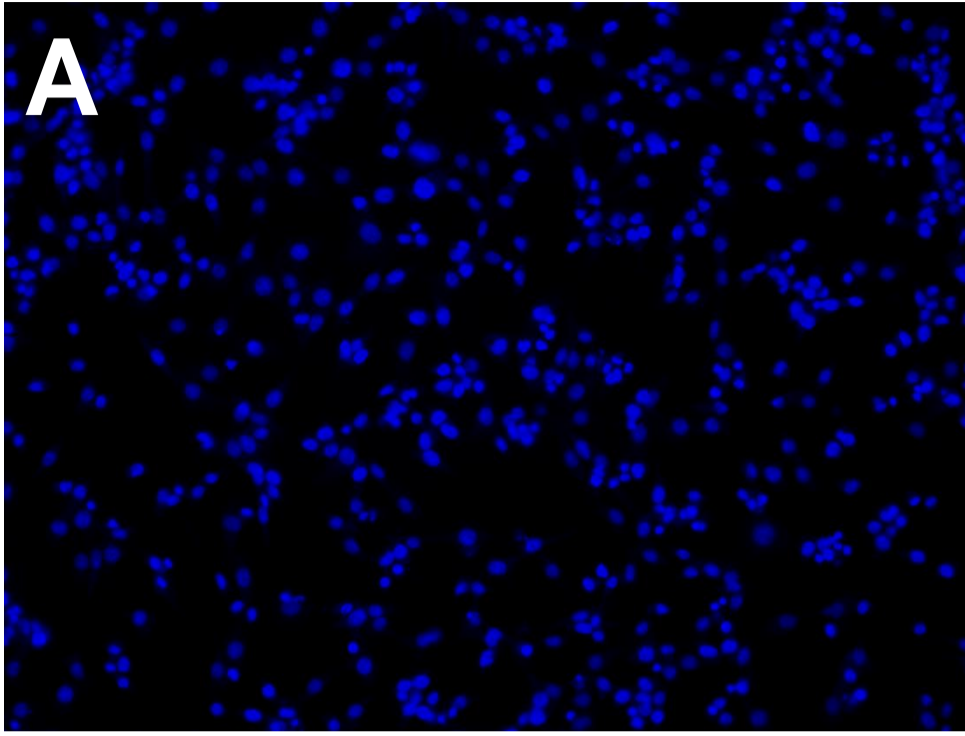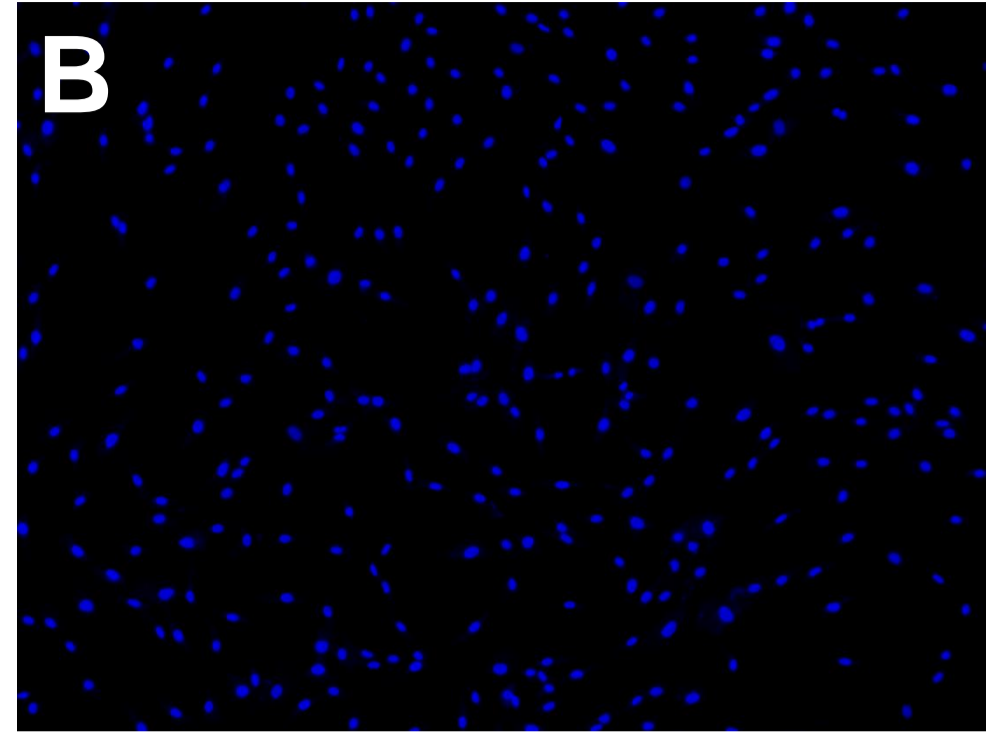

GFAP

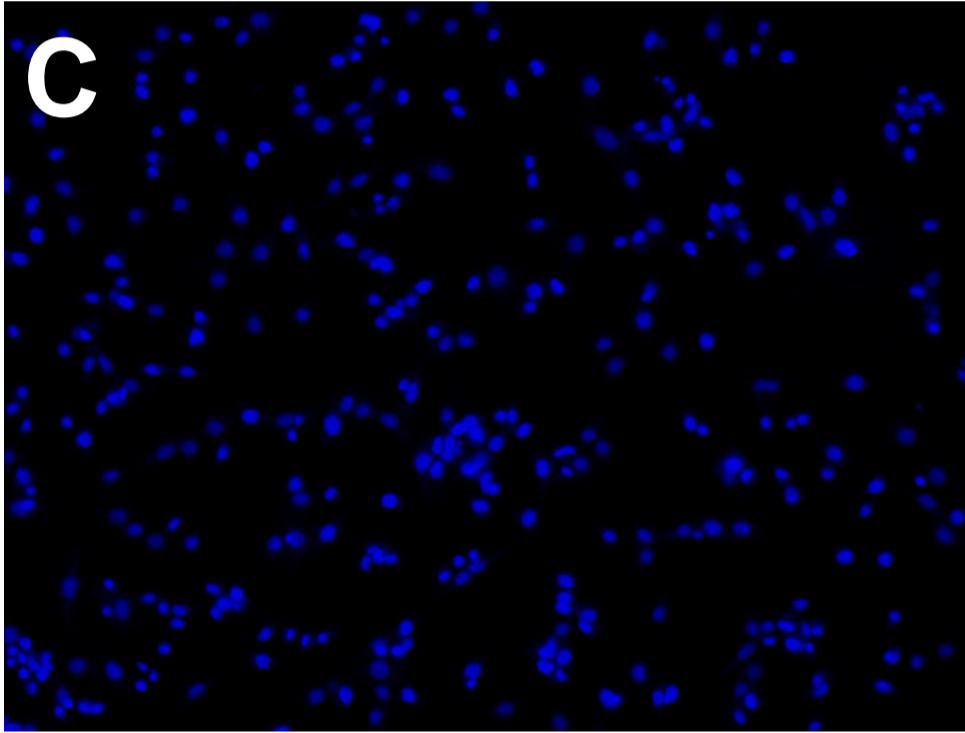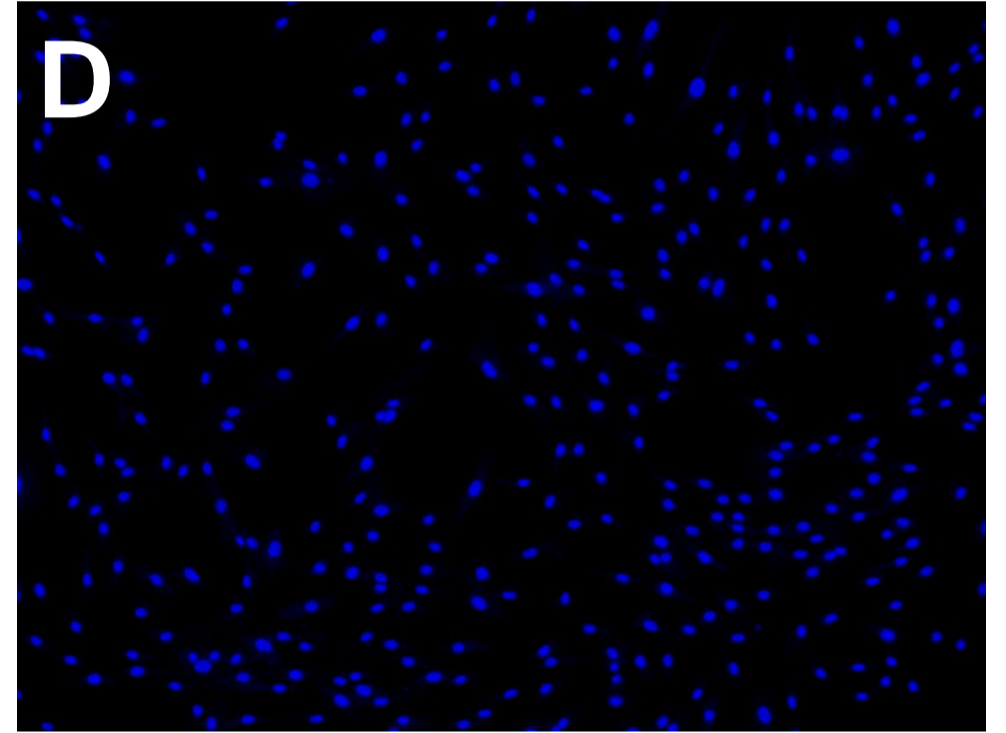

p75

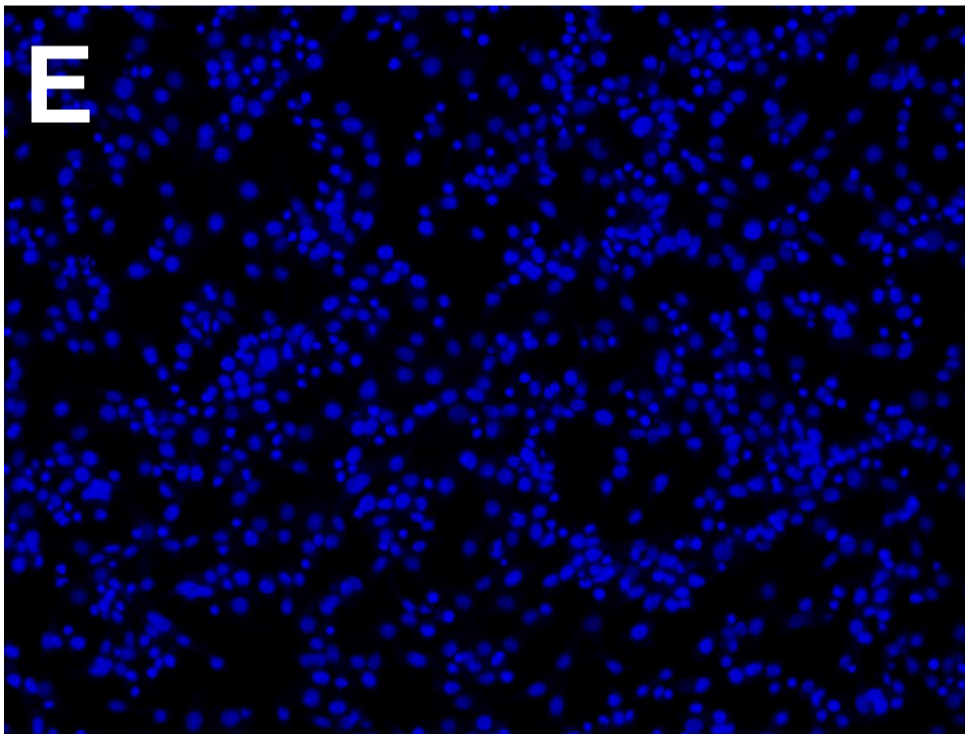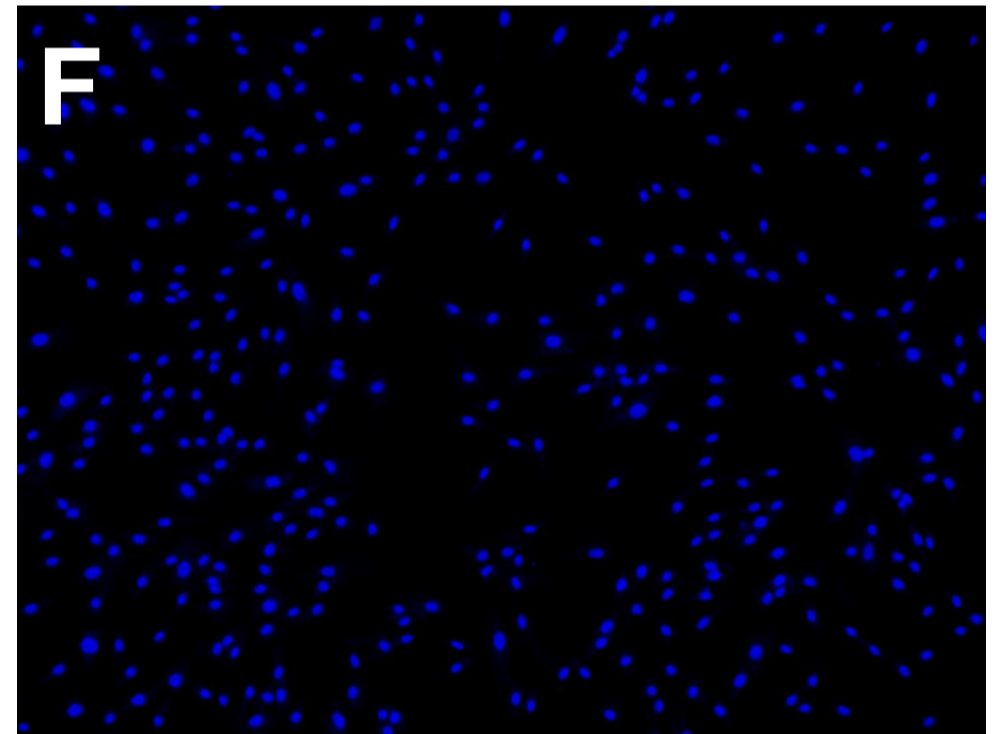

S100 $\beta$

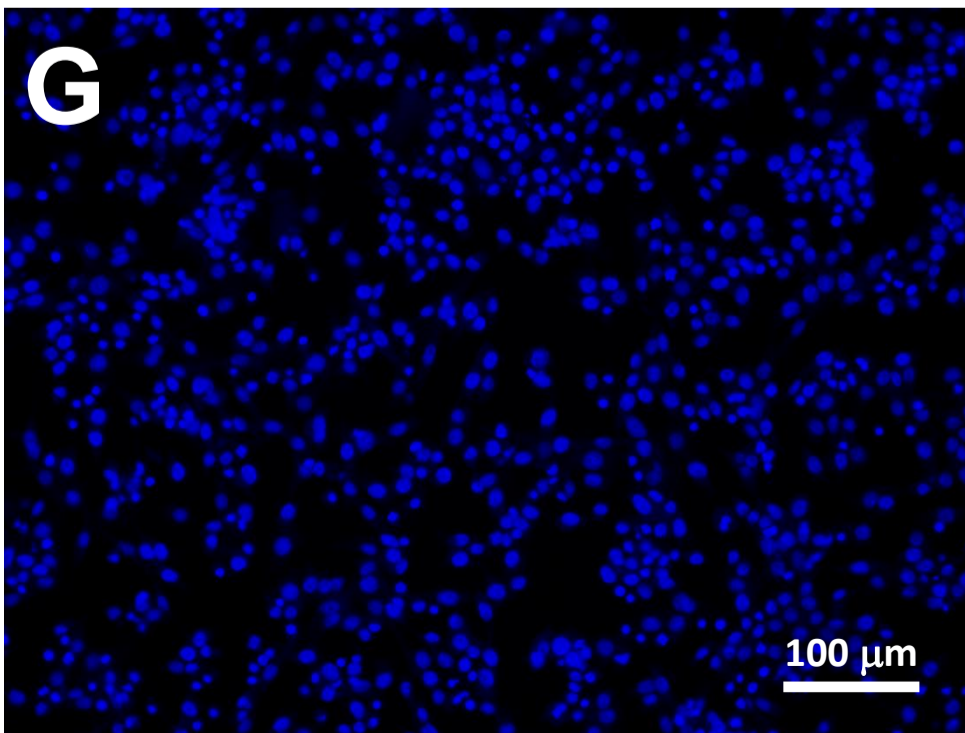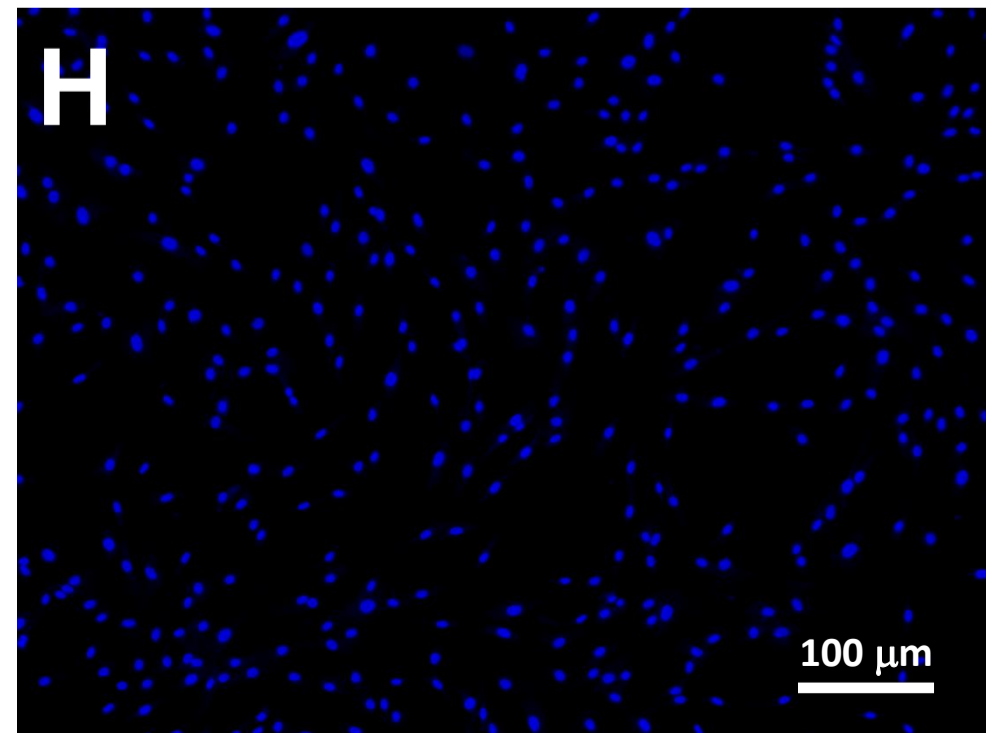

Supplement: Additional file 3: — Figure S3. Negative controls for neural marker immunocytochemistry. Immunocytochemistry performed on 3T3 cells and human BMSCs as negative controls against the neuronal marker Tuj-1 (A, B) and glial markers GFAP (C, D), p75 (E, F), and S100β (G, H) demonstrated an absence of expression. (PDF 478 kb) [file 13287_2016_409_MOESM3_ESM.pdf]

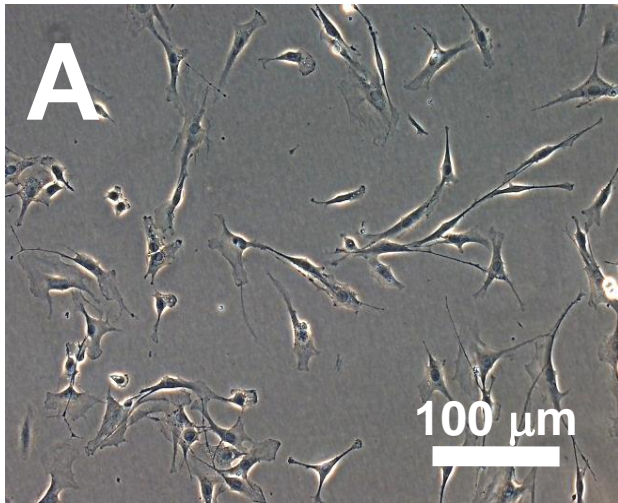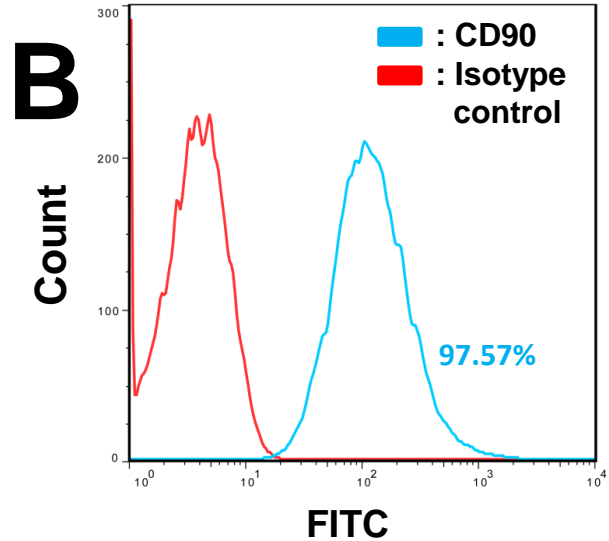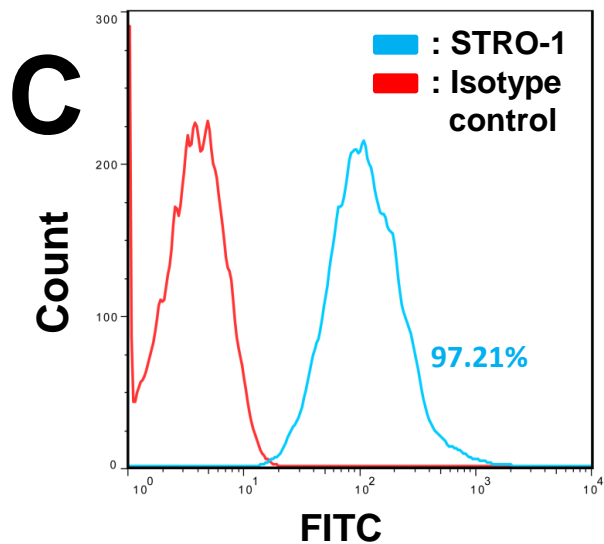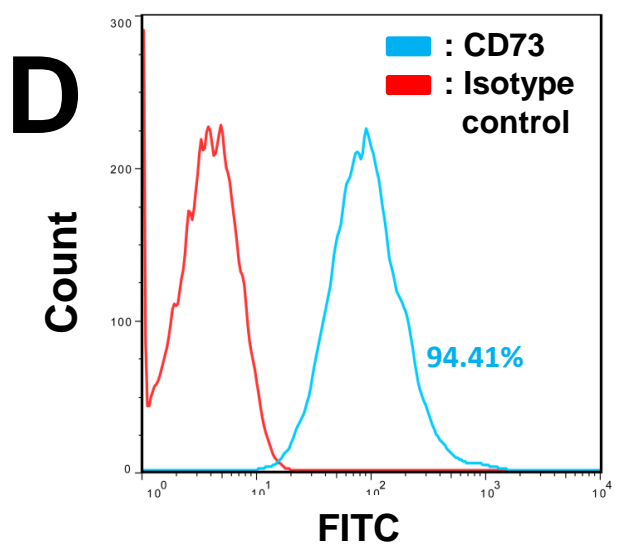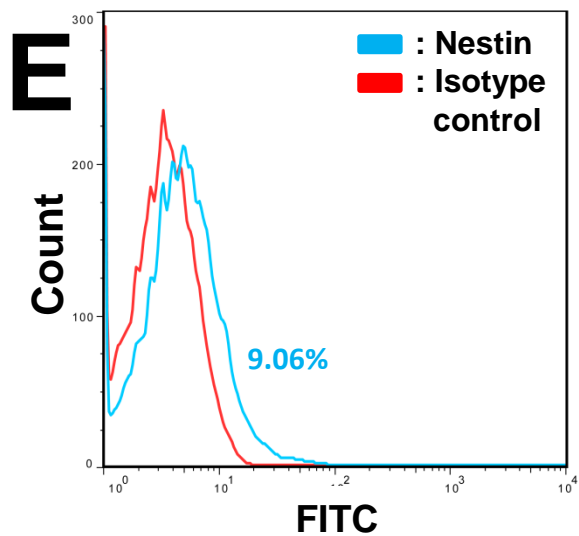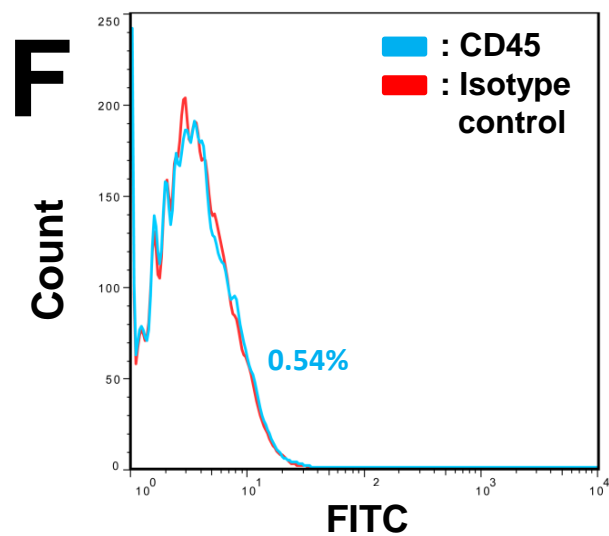

Supplement: Additional file 4: — Figure S4. Flow cytometry analysis of rat BMSCs. Rat BMSCs (A) between passage number 5 and 8 were analyzed by flow cytometry. In all panels, isotype controls are represented by red lines, while immunopositivity for respective cell surface markers are represented by blue lines. Percentages of positive cells as shown within individual panels are representative of one sample. Immunopositivity for CD90, STRO-1, and CD73 in isolated rat BMSCs were 95.70 ± 1.21 %, 95.91 ± 1.86 %, and 92.80 ± 1.46 %, respectively (B–D). Immunopositivity for nestin was 11.31 ± 1.25 % (E). Immunopositivity towards the hematopoietic progenitor marker CD45 was negligible (F). Mean ± SD, n = 4. (PDF 288 kb) [file 13287_2016_409_MOESM4_ESM.pdf]
